# Actin Cytoskeleton and Integrin Components Are Interdependent for Slit Diaphragm Maintenance in *Drosophila* Nephrocytes

**DOI:** 10.3390/cells13161350

**Published:** 2024-08-14

**Authors:** Megan Delaney, Yunpo Zhao, Joyce van de Leemput, Hangnoh Lee, Zhe Han

**Affiliations:** 1 Center for Precision Disease Modeling, Department of Medicine, University of Maryland School of Medicine, 670 West Baltimore Street, Baltimore, MD 21201, USA; 2 Division of Endocrinology, Diabetes and Nutrition, Department of Medicine, University of Maryland School of Medicine, 670 West Baltimore Street, Baltimore, MD 21201, USA

**Keywords:** actin cytoskeleton, integrin, *Drosophila*, nephrocyte, slit diaphragm, lacunar channel, piezo

## Abstract

In nephrotic syndrome, the podocyte filtration structures are damaged in a process called foot process effacement. This is mediated by the actin cytoskeleton; however, which actins are involved and how they interact with other filtration components, like the basement membrane, remains poorly understood. Here, we used the well-established *Drosophila* pericardial nephrocyte—the equivalent of podocytes in flies—knockdown models (RNAi) to study the interplay of the actin cytoskeleton (Act5C, Act57B, Act42A, and Act87E), alpha- and beta-integrin (basement membrane), and the slit diaphragm (Sns and Pyd). Knockdown of an actin gene led to variations of formation of actin stress fibers, the internalization of Sns, and a disrupted slit diaphragm cortical pattern. Notably, deficiency of *Act5C*, which resulted in complete absence of nephrocytes, could be partially mitigated by overexpressing *Act42A* or *Act87E*, suggesting at least partial functional redundancy. Integrin localized near the actin cytoskeleton as well as slit diaphragm components, but when the nephrocyte cytoskeleton or slit diaphragm was disrupted, this switched to colocalization, both at the surface and internalized in aggregates. Altogether, the data show that the interdependence of the slit diaphragm, actin cytoskeleton, and integrins is key to the structure and function of the *Drosophila* nephrocyte.

## 1. Introduction

In kidney diseases, like nephrotic syndrome, the highly specialized podocytes of the kidney glomerulus are damaged or even lost. In healthy kidneys, the neighboring podocytes form intercalating foot processes at the outer surface of glomerular capillaries. These cellular junctions provide the scaffolding for the slit diaphragm (SD), which together with the capillary fenestrated endothelial cells and the glomerular basement membrane, forms the filtration structure [1,2]. The cytoskeleton guides the transformation of simple columnar epithelial cells into fully differentiated podocytes with foot processes, is key to podocyte polarization, and facilitates adhesion of the foot process to the basement membrane. The latter is required for SD formation and enables signaling responses to external stimulation [3,4,5].

Cytoskeleton components are optimized for each of the podocyte’s compartments. They consist of microtubules and intermediate filaments in the bigger primary processes that lead from the cell body, whereas in the thinner secondary foot, processes mainly contain actin filaments [6,7,8]. Foot process effacement, which disrupts filtration structures and function, is a common feature of nephropathy and some kidney diseases [9,10,11,12]. Given the importance of the cytoskeleton in providing podocytes structure, it is not surprising that besides genes that encode SD and basement membrane components, mutations have been found in genes related to the actin cytoskeleton and its regulatory factors [13,14,15,16,17,18]. The latter include actin-binding proteins and integrins, a membrane component known for its role in cell adhesion [16,19,20,21,22,23,24,25,26,27,28,29,30,31]. Systematic studies in *Drosophila* have provided functional validation for the role of many of these genes in nephrotic syndrome [14,15].

*Drosophila* nephrocytes are the structural and functional equivalent of mammalian podocytes [2]. Like podocytes, nephrocytes have SD and basement membrane filtration structures. In nephrocytes, the SD is formed intracellularly, spanning lacunar channel openings [2,14]. Low molecular weight molecules are reabsorbed by endocytosis at the lacunar channel membranes, thus filtering the hemolymph, the insect equivalent of blood [2,14]. In fact, ~85% of genes associated with nephrotic syndrome have conserved functions in *Drosophila* nephrocytes, including those encoding components of the SD and cytoskeleton [14,15], which has made flies one of the key animal models to gain understanding of the cellular and molecular mechanisms in kidney diseases and development [32]. Here, we used *Drosophila* nephrocytes to investigate the relationship between SD components, the actin cytoskeleton, and integrins. Our findings reveal the importance of the cytoskeleton for the formation and maintenance of the SD and indicate supporting yet distinct roles for the different actin genes expressed in nephrocytes. Furthermore, we demonstrate the interdependence of the SD, actin cytoskeleton, and integrins in nephrocytes.

## 2. Materials and Methods

### 2.1. Drosophila Husbandry

Fly lines were maintained on a standard diet (Meidi Laboratories, Potomac, MD, USA), at 25 °C under a 12 h:12 h light:dark cycle. We used two independent UAS-RNAi lines for silencing each gene. *Drosophila* stocks were obtained from the Bloomington Drosophila Stock Center (BDSC, IN): UAS-*Act5C-*IR (BDSC_42651), UAS-*Act42A*-IR (BDSC_50625), UAS-*Act57B*-IR (BDSC_31551), UAS-*Act87E*-IR (BDSC_42652), UAS-*Act5C*-*GFP* (BDSC_9258), UAS-*Act42A*-*GFP* (BDSC_9251), UAS-*Act57B*-*GFP* (BDSC_9256), UAS-*Act87E*-*GFP* (BDSC_9249), UAS-*GFP*-*Piezo* (BDSC_58773), UAS-*mew*-IR (BDSC_44553), UAS-*mys*-IR (BDSC_27735), UAS-*sns*-IR (BDSC_64872), UAS-*pyd*-IR (BDSC_28920), tub-Gal80^ts^/TM2 (BDSC_7017), tub-Gal80^ts^; TM2/TM6B (BDSC_7108), *w*^1118^ (BDSC_3605); and the Vienna Drosophila Resource Center (VDRC, Vienna, Austria): UAS-*Act5C*-IR (VDRC_101438), UAS-*Act42A*-IR (VDRC_104731), UAS-*Act57*B-IR (VDRC_102129), and UAS-*Act87E*-IR (VDRC_102480). The following are in-house fly stocks: *Hand-GFP*, *hs*-*Flp^122^*; UAS-*Flp*; *Act5C*>*stop*>Gal4, UAS-*GFP* [33]; and If/CyO.

### 2.2. Generation of Drosophila Klf15-Gal4 Line

To generate the *Klf1*5-Gal4 transgenic line, a 2.1 kb *Klf15* promoter region was PCR amplified and cloned into the pPTGAL vector between the EcoRI and BamHI restriction sites. The plasmid was sequence-verified. Microinjection was performed by Rainbow Transgenic Flies (CA).

### 2.3. Knock-In mRuby3 Tag at the C-Terminal of Sns

Transgenic *sns*-mRuby3 flies carry an mRuby3 tag at the C-terminal of endogenous Sns. The line was commercially generated by FunGene (Jiangsu, China). SnsKI-sg1 and SnsKI-sg2 gRNAs were generated using in vitro transcription (T7 RiboMAX kit; P1320; Promega, WI, USA). The transcripts were purified by phenol-chloroform extraction and isopropanol precipitation. Cas9 mRNA in vitro transcription was carried out using plasmid MLM3613 (plasmid 42251; Addgene, MA, USA), while the pBluescript SK vector (pBS) was used as the backbone for donor plasmid construction. Using genomic DNA of the injection stock, the 5′ and 3′ homology arms were amplified and linked to the pBS backbone (Gibson assembly kit; E2611L; NEB, MA, USA). This pBS-*sns*-arm was linearized by PCR and linked to the mRuby3 cassette (Gibson assembly kit; E2611L; NEB, MA, USA), resulting in the final donor construct: pBS-*sns*-mRuby3. The gRNA, Cas9 mRNA, and donor pBS-*sns*-mRuby3 were injected into *w*^1118^ flies. In house, PCR was performed to validate the mRuby3 insertion.

### 2.4. Immunochemistry

Female adult (1-day-old) flies were rinsed in 95% ethanol and transferred to 1xPBS. The *Drosophila* abdomen was cut open to remove intestine, Malpighian tubules, fat bodies, and ovaries. The resulting dorsal cuticles of the abdomen (with nephrocytes and heart tube attached) were fixed in 4% PFA for 1 h at room temperature. The specimens were washed three times in 1xPBST for 15 min (0.2% Triton-x 100 in 1xPBS) and then blocked in blocking buffer (1% bovine serum albumin in 1xPBST) for 1 h. Primary antibodies were diluted in the blocking buffer and incubated overnight at 4 °C and then washed in 1xPBST three times for 15 min each, followed by incubation with secondary antibodies for 2 h at room temperature. The samples were then incubated with phalloidin (Alexa Fluor 488 phalloidin; 1:100; A12379; Invitrogen, CA, USA) diluted in blocking buffer for 4 days at 4 °C. The following antibodies were used: Mouse monoclonal anti-Pyd (1:100; RRID:AB_2618043; Developmental Studies Hybridoma Bank, IA), anti-Mys (1:100; CF.6G11, RRID_ AB_528310; Developmental Studies Hybridoma Bank, IA), and goat anti-mouse Alexa Fluor 488 (1:500; A11011, RRID:AB_143157; Invitrogen, CA, USA). DAPI (0.5 mg/mL in PBST; D1306; Thermo-Fisher-Scientific, MA, USA) was used to visualize the nuclei. Following antibody incubations, the samples were washed in 1xPBST three times for 15 min each, once with 1xPBS for 15 min, and then mounted using Vectashield antifade mounting media (H-1000; Vector Laboratories, CA, USA). Images were obtained using a ZEISS LSM900 confocal microscope with 63× Plan-Apochromat 1.4 N.A. oil objective in Airyscan mode and ZEN blue edition (version 3.0) acquisition software. Representative images are shown in the figures. Five flies per genotype were analyzed, of which three representative images were taken and used for quantification and figures. For quantitative comparison of fluorescence intensities, settings for the control were chosen to avoid oversaturation (using range indicator in ZEN blue) and then applied across images for all samples within an assay. Image J [34] was used for image processing (version 2.9.0/1.53t; National Institutes of Health, MD, USA).

### 2.5. RNA-Seq Embryonic and Adult Nephrocytes

Embryonic RNA-seq data (GSE168774; can also be accessed through the Single Cell Portal at https://singlecell.broadinstitute.org/, accessed on 8 August 2024) was carried out previously [35]. For the adult low input RNA-seq (GSE266297), the flies (*Hand*-GFP; *Klf15*-Gal4, UAS-RFP/CyO) were maintained on a standard diet (Meidi Laboratories, MD, USA), at 25 °C under a 12 h:12 h light:dark cycle. Nephrocytes from adult flies (4-day-old females; 3 replicates of 200 flies each) were dissected in artificial hemolymph (108 mM Na^+^, 5 mM K^+^, 2 mM Ca^2+^, 8 mM MgCl_2_, 1 mM NaH_2_PO_4_, 4 mM NaHCO_3_, 10 mM sucrose, 5 mM trehalose, and 5 mM HEPES; pH 7.1) at room temperature. Large, GFP- and RFP-positive cells were sorted by flow cytometry (BD Aria II; University of Maryland Greenebaum Comprehensive Cancer Center Flow Cytometry Shared Service). Following this, RNA was collected and cDNA libraries were prepared using the SuperScript IV Single Cell/Low-Input cDNA PreAmp Kit (Thermo-Fisher-Scientific, MA, USA). Then, sequencing on a NovaSeq platform (Illumina, CA, USA) was carried out by Psomagen (Rockville, MD, USA). We mapped the short reads on to the *Drosophila* genome using STAR aligner 2.7.5c [36] and then quantified the RNA-seq reads using RSEM 1.3.3 [37] based on *Drosophila* gene annotation 6.28 from FlyBase (Berkeley Drosophila Genome Project (BDGP) Release 6).

### 2.6. Nephrocyte Number and Size Quantifications

Nephrocytes from 1-day-old adult female flies were dissected in 1xPBS, followed by fixation (1 h) in 4%PFA, and imaged using a ZEISS LSM900 confocal microscope 20× Plan-Apochromat 0.8 N.A. air objective. The number of nephrocytes present in the image were then counted, and cell sizes were determined using the area measurement function using Fiji software [34] (version 2.9.0; National Institutes of Health, MD, USA). Five flies per genotype were analyzed, of which three representative images were taken and used for quantification and figures.

### 2.7. 10 kD Dextran or 70 kD Dextran Uptake

Nephrocyte functional assays were performed ex vivo at room temperature. *Drosophila* females (1-day-old adults) were dissected in Schneider’s Drosophila Medium (Thermo-Fisher-Scientific, MA, USA) and then incubated for 20 min in a 10 kD Texas Red-dextran solution (0.05 mg/mL; D1828; Invitrogen, CA, USA) in Schneider’s Drosophila Medium (Thermo-Fisher-Scientific, MA), or alternatively, incubated for 1 min in a 70 kD Texas Red-dextran solution (0.25 mg/mL; D1864; Invitrogen, CA, USA) in Schneider’s Drosophila Medium (Thermo-Fisher-Scientific, MA, USA). Following dextran uptake, the specimens were washed with Schneider’s Drosophila Medium (Thermo-Fisher-Scientific, MA) twice and then fixed using 4% paraformaldehyde (PFA) for 60 min. Finally, the fixed specimens were washed three times for 5 min each with 1× phosphate buffered saline (1xPBS; pH 7.4) and mounted using Vectashield antifade mounting medium (H-1000; Vector Laboratories, CA, USA). Images were obtained using a ZEISS LSM900 confocal microscope with 20× Plan-Apochromat 0.8 N.A. air objective and ZEN blue edition (version 3.0) acquisition software. Five flies per genotype were analyzed, of which three representative images were taken and used for quantification and figures. For quantitative comparison of fluorescence intensities, settings for the control were chosen to avoid oversaturation (using range indicator in ZEN blue) and then applied across images for all samples within an assay. Image J [34] was used for image processing (version 2.9.0/1.53t; National Institutes of Health, MD, USA).

### 2.8. Sns-mRuby3 and Phalloidin Quantifications

Since both *Klf15*-Gal4 and *sns*-mRuby3 are located on the 2nd chromosome, *Klf15*-Gal4 female virgin flies were crossed with *sns*-mRuby3 male flies, the progeny female virgins were crossed with If/CyO to create a stable stock of [*Klf15*-Gal4, *sns*-mRuby3/CyO]. These female virgins were then crossed with *w*^1118^, *Piezo*-IR, *Act5C*-IR #1, *Act5C*-IR #2, *Act42A*-IR #1, *Act42A*-IR #2, *Act57B*-IR #1, *Act57B*-IR #2, *Act87E-*IR #1, *Act87E*-IR #2, *mew-*IR (encodes α-integrin subunit), or *mys*-IR (encodes β-integrin subunit). The nephrocytes from 1-day-old females were then stained according to the immunochemistry protocol detailed above and imaged using the ZEISS LSM900 confocal microscope with 63× Plan-Apochromat 1.4 N.A. oil objective under Airyscan mode and ZEN blue edition (version 3.0) acquisition software. For stress fibers and SD internalization, the number of nephrocytes with stress fibers (any cytoplasmic actin structure, based on phalloidin stain) were manually counted. Nephrocytes numbers analyzed were 13 for control, 39 for *Act42*-RNAi, 52 for *Act57B*-RNAi, and 51 for *Act87E*-RNAi flies. To determine internalized Sns (based on the presence of distinct cytosplasmic Sns-mRuby) nephrocytes were manually counted: 24 for control, 51 for *Act42A*-RNAi, 36 for *Act57B*-RNAi, and 33 for *Act87E*-RNAi flies. For quantitative comparison of fluorescence intensities, settings for the control were chosen to avoid oversaturation (using range indicator in ZEN blue) and then applied across images for all samples within an assay. Five flies per genotype were analyzed, of which three representative images were taken and used for quantification. Image J [34] was used for image processing (version 2.9.0/1.53t; National Institutes of Health, MD, USA). The average number of slit diaphragm lines (visualized by Sns-mRuby) were depicted using Image J [34]. Nephrocytes from RNAi (-IR) flies were compared to SD patterns in control nephrocytes.

### 2.9. Tissue Mosaic Analysis

Flp-out clone [38] induction was performed as described previously [33]. In brief, *hs*-*Flp^122^*; UAS-*Flp*; *Act5C*>*stop*>Gal4, UAS-*GFP* female virgins were crossed with α-*integrin*-IR and β-*integrin*-IR males. The embryos were collected within an eight-hour time window. At 24 h after larval hatching, a 10-min heat shock was performed in a 37 °C water bath, following which the larvae were maintained at 25 °C. One-day-old female adults were subjected to 10 kD dextran functional assay (described above); GFP-positive nephrocyte clones and their neighboring nephrocytes were analyzed.

### 2.10. TARGET Assay

*Klf15*-Gal4, *sns*-mRuby3 virgin female flies were initially crossed with *tub*-Gal80^ts^/TM2, located on chromosome 3. These virgin female flies were then crossed with *Klf15*-Gal4;*sns*-mRuby/*Act5C-*IR males, resulting in [*Klf15*-Gal4, *sns*-mRuby3/+; *tub*-Gal80^ts^/*Act5C-*IR] flies. TARGET [39,40]. These flies were kept at 18 °C until their offspring had reached 1 day of age. This allowed for the Gal80 to be activated, which repressed Gal4 activity. The 1-day-old adults were then transferred to 29 °C for either one or two days; this temperature Gal80 was inactivated, allowing the Gal4 to become active and knock down the *Act5C* gene in the nephrocytes. Following this temperature switch, the flies were assayed and imaged as described above.

### 2.11. Actin Protein Sequence Comparisons

Protein sequences were obtained from FlyBase [41,42] (version 2023_05: Dmel 6.54) for *Drosophila* and UniProt [43] (release 2023_04) for human and then aligned using the Multiple Sequence Alignment (MSA) package [44] in R [45] (version 4.3.1) using the Clustal Omega algorithm [46]. Phylogenetic analyses were based on protein sequences and carried out using the Neighbor-Joining Tree Estimation function from the Analyses of Phylogenetics and Evolution (APE) package [47] in R [45] (version 4.3.1).

### 2.12. Statistical Analysis

Fiji software [34] (version 2.9.0; National Institutes of Health, MD, USA) was used to process the confocal images and to quantify the relative fluorescence intensity. The data sets were tested for normality using the Shapiro–Wilk test. Normally distributed data were analyzed by a two-tailed Student’s *t*-test, a one-way ANOVA corrected with Tukey, or by a two-way ANOVA with Sidak correction. Non-normally distributed data were analyzed by a Mann–Whitney U test or a Kruskal–Wallis H test. *p* < 0.05 was considered significant. The data sets were plotted using GraphPad Prism9 software (version 9.5.1). The figures were arranged using Adobe Illustrator software (version 2022 26.2.1).

## 3. Results

### 3.1. The Actin Cytoskeleton Surrounds the Lacunar Channels

**Figure 1:**
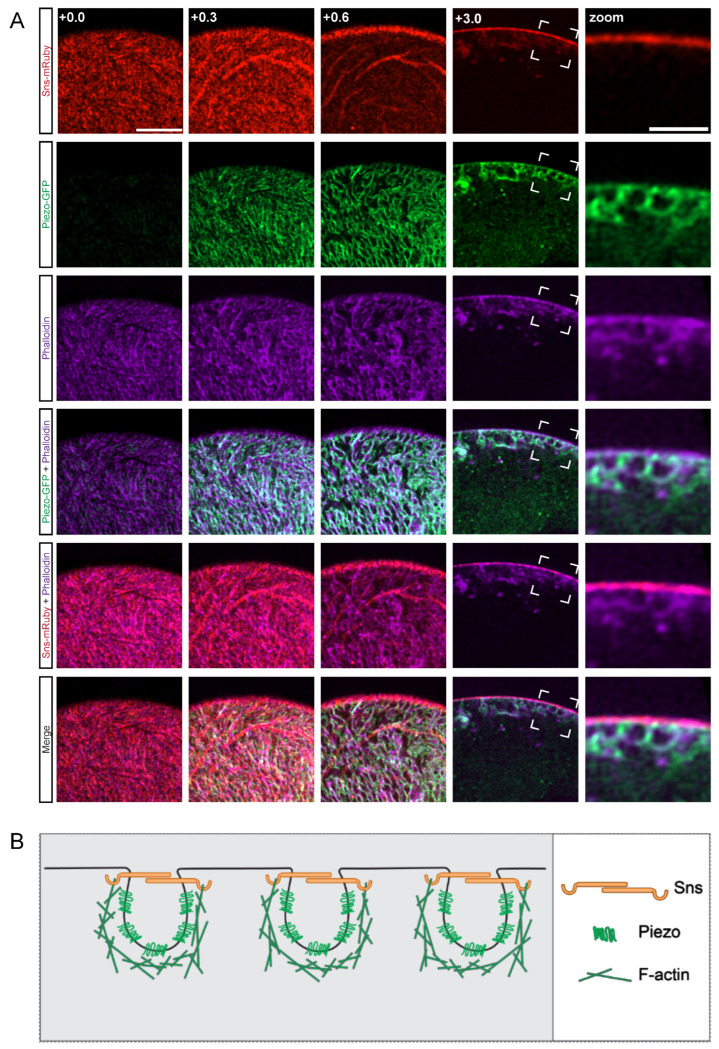
**Actin cytoskeleton encloses Piezo below the SD filtration structure.** (**A**) Shown are representative confocal images of *Klf15*-Gal4, *sns*-mRuby3/UAS-GFP-*Piezo* nephrocytes (1-day-old, female). The columns show the cortical and subcortical regions of z-stacks separated by 0.3 µm (+0.0; +0.3; +0.6) and the medial view (+3.0). Box indicates the magnified image (zoom) to show the plane at which actin (phalloidin), Piezo, and sticks and stones (Sns-mRuby3) intersect directly beneath the membrane surface. Sns-mRuby3 is in red, Piezo-GFP is in green, Phalloidin is in purple. Scale bars: (cortical/subcortical) 5 µm; (zoom) 2 µm. (**B**) Proposed model depicting the localization and interaction of the actin cytoskeleton (F-actin), the SD (Sns), and the lacunar channel (lined by Piezo) in a typical nephrocyte.

First, we set out to determine the relative localization of the cytoskeleton to the membrane, SD, and invaginated lacunar channel filtration structures, as the actin cytoskeleton is a highly dynamic structure in nephrocytes [48]. For this, we used our previously generated transgenic fly line (*sns*-mRuby) that allows for the endogenous visualization of sticks and stones (Sns), the *Drosophila* homolog of the mammalian SD marker nephrin. We found *Piezo*-GFP to be an effective marker of the nephrocyte lacunar channels. Expression of Sns-mRuby and Piezo-GFP were controlled by the nephrocyte-specific Gal4 driver *Krupppel-like factor 15* (*Klf15*) to study their localization along with the actin cytoskeleton (visualized by phalloidin stain) (Figure 1A). Imaging at different planes revealed that in typical nephrocytes the actin cytoskeleton wraps around Piezo, essentially cupping the lacunar channel, and partially co-localized with Sns (Figure 1A). These images suggest that all three elements together are important to form the structure of the nephrocyte SD (Figure 1B).

### 3.2. Silencing Actin Genes Results in Variation in Nephrocyte Number, Size, and Function

**Figure 2:**
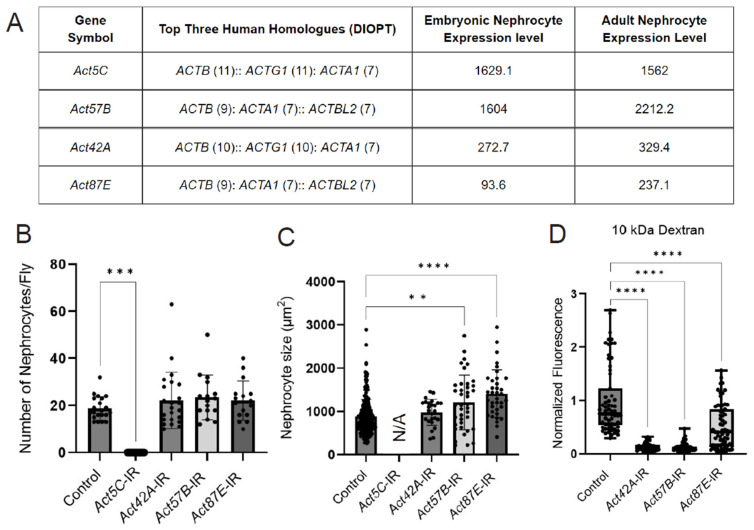
**Knockdown of nephrocyte actin genes results in disrupted nephrocyte morphology and function.** (**A**) Table displays RNA-seq data for actin genes in typical *Drosophila* nephrocytes (GSE168774; GSE266297), ranked by expression level in adult nephrocytes. DIOPT, DRSC integrated ortholog prediction tool (version 9.1) [49] to identify homologs among species. Embryonic nephrocyte expression levels given in counts per million (CPM). Adult nephrocyte expression levels provided in transcripts per million (TPM) and ranking out of 15,000 genes. (**B**) Quantification of nephrocyte number. Note: no nephrocytes were observed in *Act5C*-IR (RNAi) flies. Statistical analysis: Kruskal–Wallis test; ***, *p* < 0.001. *Klf15*-Gal4 was used as a control, which also applies in panels (**C**,**D**). (**C**) Quantification of nephrocyte size in 1-day-old female flies (see (**C**), for details on flies). Statistical analysis: Kruskal–Wallis test; **, *p* < 0.01; ****, *p* < 0.0001. Note, since no nephrocytes were left in *Act5C*-IR (RNAi) flies, cell size could not be determined. (**D**) Quantification of 10 kDa dextran uptake assays in nephrocytes from 1-day-old female flies (see Figure S1, for details on flies). Statistical analysis: Kruskal–Wallis test; ****, *p* < 0.0001.

The *Drosophila* genome encodes six different actin proteins; we detected four in nephrocytes: *Act5C* and *Act57B* were highly expressed, and *Act42A* and *Act87E* were moderately expressed (based on embryonic RNA-seq [35] and adult low input RNA-seq data) (Figure 2A). We used RNAi knockdown to investigate the function of the individual actin genes in nephrocytes. Knockdown of *Act5C* resulted in the complete loss of nephrocytes, whereas no significant changes in nephrocyte numbers were observed when the other actin genes were silenced (Figure 2B, Supplementary Figure S1). However, nephrocyte size was significantly increased following *Act57B* or *Act87E* knockdown (Figure 2C). In addition, functional assays showed significantly reduced uptake of 10 kDa dextran in nephrocytes deficient in *Act42A*, *Act57B*, or *Act87E* (Figure 2D). Together these data show that all four nephrocyte-expressed actin genes are important for structure and function. However, the extent of damage due to deficiency is different for each actin, with nephrocytes completely absent following *Act5C* knockdown, indicating potentially divergent roles within nephrocytes.

### 3.3. Knockdown of Actin in Nephrocytes Results in Distinct Structural Defects

**Figure 3:**
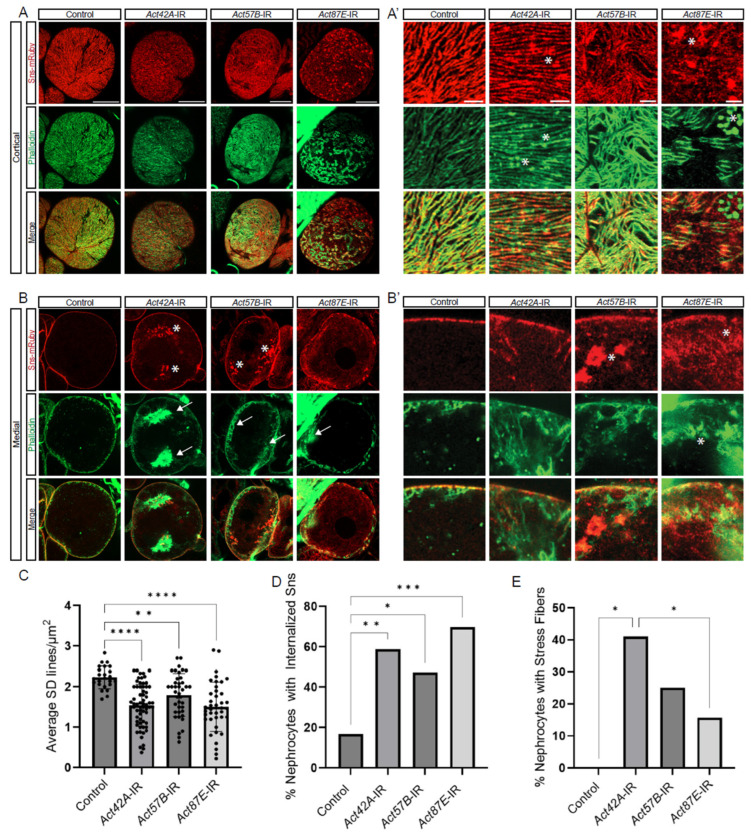
**Distinct roles for different actin genes in maintaining the cytoskeleton and SD structures.** (**A**,**A’**,**B**,**B’**) Fluorescent confocal images of Control, *Act42A*-IR (RNAi), *Act57B*-IR, and *Act87E*-IR nephrocyte SD (Sns-mRuby) and actin cytoskeletons (phalloidin). Sns-mRuby3 is in red, Phalloidin is in green. Whole cell cortical views (**A**) alongside magnified cortical views (**A’**); whole cell medial views (**B**) alongside magnified medial views (**B’**). Asterisks (*) indicate aggregations, arrows indicate actin stress fibers. Scale bars: (**A**) 20 µm; (**A’**) 5 µm; (**B**) 20 µm; (**B’**) 5 µm. (**C**) Graph shows SD cortical density based on the average Sns-mRuby fluorescent peaks. Values reflect the number of SD lines per cortical region (µm^2^). Five flies were collected per genotype, in which 3 nephrocytes were analyzed. Statistical analysis: Kruskal–Wallis test; **, *p* < 0.01; ****, *p* < 0.0001. (**D**) Quantification of internalized sticks and stones (Sns-mRuby) based on the medial plane. Values represent percent nephrocytes with visible internalized Sns of total nephrocytes for that genotype. Nephrocyte numbers analyzed were 13 for control, 39 for *Act42*-RNAi, 52 for *Act57B*-RNAi, and 51 for *Act87E*-RNAi flies Statistical analysis: Kruskal–Wallis test; *, *p* < 0.05; **, *p* < 0.01; ***, *p* < 0.001. (**E**) Quantification of actin stress fibers based on the medial plane. Values represent the percent nephrocytes with visible actin stress fibers of total nephrocytes for that genotype. Nephrocyte numbers analyzed were 24 for control, 51 for *Act42A*-RNAi, 36 for *Act57B*-RNAi, and 33 for *Act87E*-RNAi flies. Statistical analysis: Kruskal–Wallis test; *, *p* < 0.05.

To investigate the structural relationship between the actin cytoskeleton and the SD, we co-labeled for Sns-mRuby (SD marker) and phalloidin (F-actin) in nephrocytes deficient in each of the three actin genes with nephrocytes remaining. At the cortical surface, *Act42A*-RNAi nephrocytes showed protein aggregates positive for both Sns and F-actin; *Act57B* knockdown led to an overall increase in F-actin accumulation, whereas *Act87E* RNAi nephrocytes displayed Sns aggregates and F-actin islands, suggesting a transportation defect (Figure 3A,A’). Aggregates are defined as circular localizations with higher fluorescent densities than the surrounding areas, indicated with asterisks. At the medial plane, *Act42A*-RNAi nephrocytes showed large stress fiber aggregates, whereas *Act57B* knockdown led to large pockets of Sns protein (Figure 3B,B’). Actin stress fibers are defined as fibrous densities in the subcortical regions of the nephrocyte in place of the actin pockets seen in controls, indicated by arrows. In most cases, Sns subcortical aggregates were seen to colocalize in the same area as actin stress fibers. Indeed, the SD density was significantly decreased when any of the actin genes were knocked down (Figure 3C). Moreover, while we found few cells with internalized Sns and none with stress fibers and among control nephrocytes, those with actin deficiencies showed significant numbers of cells with internalized Sns aggregates and actin stress fiber formation (Figure 3D,E). Notably, the percentage of nephrocytes with stress fibers was significantly higher in *Act42A*-IR than in *Act87E*-IR nephrocytes (Figure 3E). These findings demonstrate critical, yet varying, structural changes in the cytoskeleton and SD filtration structure of nephrocytes deficient in *Act42A*, *Act57B*, or *Act87E*, which are likely responsible for the reduced uptake function.

### 3.4. Temporal-Dependent Knockdown of Act5C Shows Significant Structural Defects

*Act5C*-RNAi resulted in complete absence of nephrocytes (Figure 2B, Supplementary Figure S1). Therefore, to study the structural relationship between the actin cytoskeleton and the SD in *Act5C*-deficient nephrocytes, we used a Gal80/Gal4 system that gives control of temporal and regional gene expression (TARGET [39,40]; Supplementary Figure S2A). Following one day of *Act5C*-deficiency, the typical Sns fingerprint-like pattern broke down, and after two days, thick subcortical stress fibers formed (Supplementary Figure S2B–D). Thus, Act5C is also required for the maintenance of the actin cytoskeleton in nephrocytes, which provides vital structural support for the SD.

### 3.5. Partial Functional Redundancy among Actin Genes in Nephrocytes

**Figure 4:**
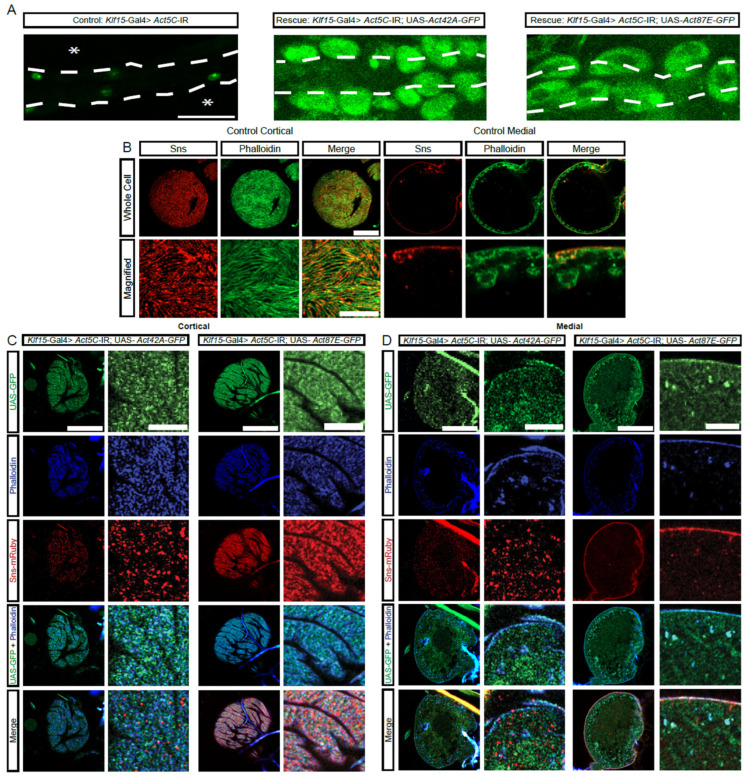
Actin from distinct protein sequence group can rescue the nephrocyte phenotype caused by deficient actin from a different group. (**A**) Representative images of heart tubes (dashed outline) and adjacent nephrocytes (1-day-old, female) from *Klf15*-Gal4, *Hand*-GFP> *Act5C*-IR flies (asterisks indicate missing nephrocytes; control) crossed with UAS-*Act42A*-GFP (group1) or UAS-*Act87E*-GFP (group 2). *Hand*-GFP, green fluorescence visualized nephrocytes. Scale bar: 50 µm. (**B**) Representative control nephrocyte with cortical and medial views. Phalloidin stains F-actin in green, Sns-mRuby3 is shown in red. Scale bars: 20 µm; (magnified) 5 µm. (**C**) Representative confocal images of the cortical surface of nephrocytes from the flies in (**A**). Phalloidin stains F-actin blue, Sns-mRuby3 is shown in red, GFP is in green. Scale bars: 20 µm; (zoom) 5 µm. (**D**) Representative confocal images of the medial plane of nephrocytes from the flies in (**A**). Phalloidin stains F-actin in blue, Sns-mRuby3 is shown in red, GFP is in green. Scale bars: 20 µm; (zoom) 5 µm.

The protein sequences for the four actin genes expressed in nephrocytes are very similar (Supplementary Figure S3A,B). However, based on their sequences, the proteins can be divided into two subgroups: group 1, *Act5C* and *Act42A*; and, group 2, *Act57B* and *Act87E* (Figure 4A,B). Notably, each group contains one actin that is highly and one that is moderately expressed in nephrocytes (Figure 2A). We next tested whether there is functional redundancy between these actin genes. Expressing either *Act42A*-GFP (group 1) or *Act87E*-GFP (group 2) in *Act5C*-RNAi flies, which have no nephrocytes, restored the nephrocytes (Figure 4A). Compared to controls (Figure 4B), nephrocytes in both rescues displayed the SD component Sns at the cortical surface, but in a more punctate distribution rather than the typical fingerprint-like pattern (Figure 4C). The *Act42A* nephrocytes showed rough Sns distribution at the surface membrane, as well as Sns protein and actin aggregates in the cytoplasm, whereas *Act87E* showed more regular Sns localization at the surface membrane and less actin accumulated in the subcortical region (Figure 4D). These findings demonstrate partial functional redundancy between the different actin genes in nephrocytes, irrespective of group or expression level, indicating divergent specialized functions.

### 3.6. Integrin Is Strongly Associated with SD Structure and Nephrocyte Function

**Figure 5:**
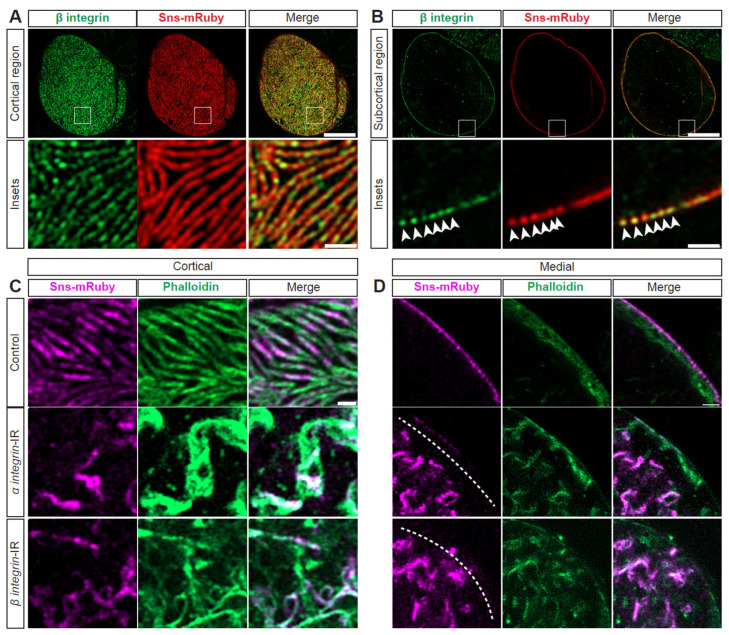
**SD structure and function depend on integrin.** (**A**) Representative confocal images of cortical surface of nephrocytes from *Klf15-*Gal4, *sns-*mRuby3/+ flies (1-day-old, female). Boxed area shown magnified below (insets). Scale bars: 10 µm; (insets) 1 µm. (**B**) Representative confocal images of the medial plane of nephrocytes from *Klf15-*Gal4, *sns-*mRuby3/+ flies (1-day-old, female). Boxed area shown magnified below (insets). Arrow heads point to the dots that show Beta-integrin and Sns-mRuby3 fluorescence. Scale bars: 10 µm; (insets) 1 µm. (**C**) Representative confocal images of the magnified cortical surface of nephrocytes from control (*Klf15-*Gal4, *sns-*mRuby3/+), *alpha-integrin*-IR (*mew* RNAi), and *beta-integrin*-IR (*mys* RNAi) flies (1-day-old, females). Dashed line outlines the nephrocyte. Scale bar: 1 µm. (**D**) Representative confocal images of the magnified medial plane of nephrocytes from control (*Klf15-*Gal4, *sns-*mRuby3/+), *alpha-integrin*-IR (*mew* RNAi), and *beta-integrin*-IR (*mys* RNAi) flies (1-day-old, females). Dashed line outlines the nephrocyte. DAPI stain used to visualize the nucleus. Scale bar: 1 µm.

Integrins have been implicated in foot process effacement [50,51] and are known to closely interact with actins [5,52]. In nephrocytes, integrin(-beta) localized at the SD with Sns (Figure 5A,B). To test functional implications, we conducted an flp-out assay that generates knockdown nephrocytes (expressing RNAi and GFP) and control nephrocytes within the same fly (Supplementary Figure S4A). Integrin (alpha/beta) knockdown resulted in significantly reduced 10 kDa dextran uptake (Supplementary Figure S4B,C)). Structurally, at the cortical surface, both the SD and actin cytoskeleton were disrupted; this was more prominent with alpha-integrin knockdown (Figure 5C, Supplementary Figure S4D). After knockdown of either integrin, the cytoplasm showed Sns and actin aggregates (Figure 5D, Supplementary Figure S4E). Together, these data show the importance of integrins for the nephrocyte SD filtration structure and function.

### 3.7. Cytoskeleton, Integrin, and SD Interdependence

**Figure 6:**
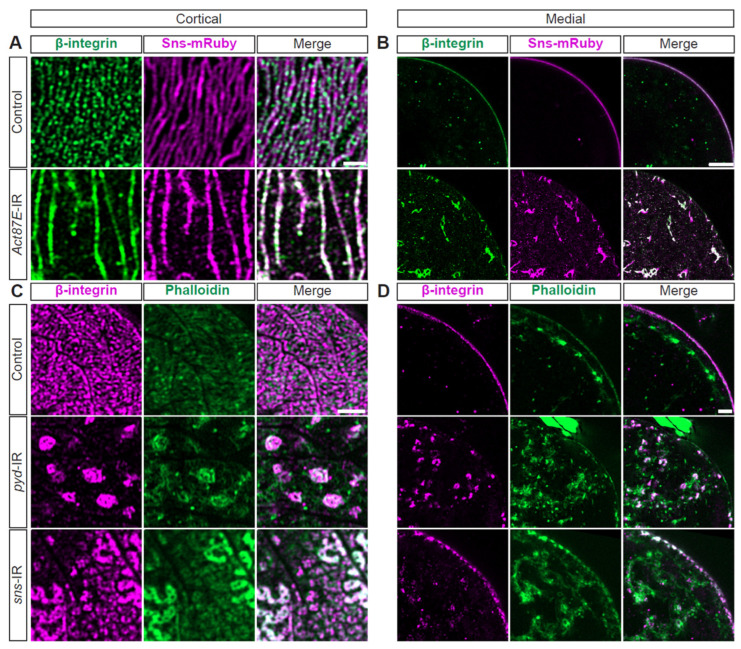
**Actin cytoskeleton, integrin, and SD structures are interdependent.** (**A**) Representative confocal images of magnified cortical surface of nephrocytes from control (*Klf15-*Gal4, *sns-*mRuby3/+) and *Act87E*-IR (RNAi) flies (1-day-old, female). Scale bars: 1 µm. (**B**) Representative confocal images of magnified medial view of nephrocytes from control (*Klf15*-Gal4, *sns*-mRuby3/+) and *Act87E*-IR (RNAi) flies (1-day-old, female). Scale bars: 1 µm. (**C**) Representative confocal images of magnified cortical surface of nephrocytes from control *(Klf15*-Gal4, *sns*-nRuby3/+), *pyd*-IR (RNAi), and *sns*-IR (RNAi) flies (1-day-old, female). Scale bars: 1 µm. (**D**) Representative confocal images of magnified medial views of nephrocytes from control *(Klf15*-Gal4, *sns*-mRuby3/+), *pyd*-IR (RNAi), and *sns-*IR (RNAi) flies (1-day-old, female). Scale bars: 1 µm.

Next, we examined the possible interdependence of actin, integrin, and the SD. Knockdown of *Act87E* (lowest expressing actin gene) resulted in a near complete overlap between beta-integrin and Sns at the SD (white fluorescence; Figure 6A, Supplementary Figure S5A), while these are co-localized in an alternating pattern in control nephrocytes (Figure 6A, Supplementary Figure S5A). Furthermore, the *Act87E*-RNAi nephrocytes contained integrin and Sns aggregates in their cytoplasm (Figure 6B, Supplementary Figure S5B). Deficiency for SD components, Sns or polychaetoid (Pyd; *Drosophila* homolog for ZO-1), resulted in integrin and actin aggregates at the cortical surface. However, a semblance of the fingerprint-like pattern was still detected following *sns*-RNAi (Figure 6C, Supplementary Figure S5C). Both showed severely disrupted localization of integrin and actin, with proteins found throughout the cytoplasm (Figure 6D, Supplementary Figure S5D). These data indicate a highly regulated system and interdependence between SD components, integrin subunits, and the actin cytoskeleton.

## 4. Discussion

**Figure 7:**
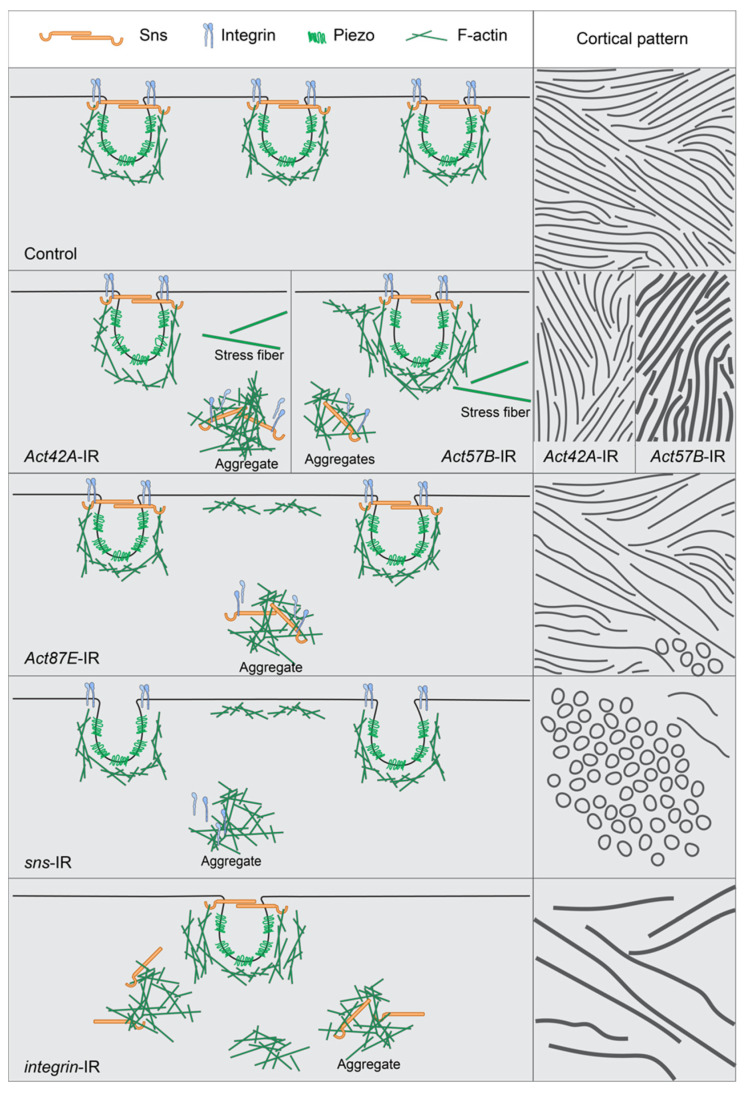
Model showing interdependence of slit diaphragm, integrin, and actin cytoskeleton when a single component is knocked down. This figure shows the normal distribution of the slit diaphragm (sns), the lacuna channel (piezo), integrin (alpha and beta subunits), and actin cytoskeleton (F-actin) in a control nephrocyte. It also models what appears to happen in the nephrocyte when either an actin, slit diaphragm, or integrin gene is no longer expressed, specifically how the remaining components accumulate cortically to create ring structures or subcortically to create aggregates or stress fibers. The left column shows a medial view while the right column shows a cortical view, with both the lines and the circles indicating the overall sns/integrin/actin interaction. Row 1 = Control, row 2 = split between *Act42A*-IR and *Act57B*-IR, row 3 = *Act87E*-IR, row 4 = *sns*-IR, and row 5 = *integrin*-IR.

This study describes the relationship between the nephrocyte SD, actin cytoskeleton, and integrins (Figure 7). Its data demonstrate that in typical nephrocytes the actin cytoskeleton supports, or cups, the entire lacunar channel, putting it in position to facilitate dynamic changes when needed. Integrin was located at the cortical surface alongside the SD marker Sns, indicating its importance for the filtration structure (Figure 7: Control). Indeed, nephrocytes deficient in either integrin subunit (alpha/beta) showed marked accumulation of actin stress fibers, internalized Sns, and loss of the distinct SD fingerprint-like pattern (Figure 7: *integrin-*IR). Nephrocytes deficient in SD components displayed actin stress fibers with internalized integrin when deficient in Pyd, but less so for Sns, as well as circular patterns of actin and integrin at the cortical surface, indicating loss of SD structural integrity (Figure 7: *sns-*IR). Finally, actin deficiency resulted in nephrocytes with cytoplasmic aggregates of SD components and integrin, while at the surface mis-localized SD components formed circular rather than a fingerprint-like pattern, evident of a severely disrupted filtration structure (Figure 7: *Act42A*-IR; *Act57E*-IR; *Act87E*-IR). In an extreme case (*Act5C*-RNAi), all nephrocytes were lost.

Together the findings indicate a high level of interdependence among the SD, actin cytoskeleton, and integrin in nephrocytes. The importance of the actin cytoskeleton and its regulators for SD formation and maintenance has been well-established in cultured human podocytes, mice, and *Drosophila* [48,53,54,55], as is its role during foot process effacement [5,8,56,57]. The reverse has been demonstrated as well, that is, Neph1 (Kirre in fly) and nephrin (Sns in fly) directly interact to facilitate actin polymerization at the plasma membrane and to recruit adapters like ZO-1 (Pyd in fly) to organize the cytoskeleton [48,58,59]. Integrins are transmembrane receptors that mediate the attachment of podocytes at the basement membrane, which is crucial to maintain the structural integrity of the glomerulus [24,50,51]. Findings in cultured human podocytes, mice, and *Drosophila* have shown that integrin activity is mediated by nephrin, and that it is important in maintaining the filtration barrier [24,60]. Furthermore, Integrin-linked kinase (ILK) acts at the intersection of the podocyte actin cytoskeleton and contact with the basement membrane [61]. ILK regulates the localization of nephrin (Sns in fly) and alpha-actinin-4 and, as such, is important in maintaining the SD and intrinsically linking integrin with the SD [22]. Notably, beta-integrin deficiency was shown to lead to proteinuria preceding disruption of the cytoskeleton, as well as podocyte detachment and apoptosis [22,62,63], whereas ectopic expression disrupted cell–cell adhesion, the cytoskeleton, and SD integrity [26]. We observed that integrin colocalized with Sns at the SD in an alternating fashion along the fingerprint-like cortical pattern. When either an SD component or an actin was deficient, the distribution changed to a near complete colocalization of integrin and Sns (Figure 6A,B). This colocalization extended into the cytoplasm, where we observed a high level of concordance between cytoplasmic aggregates containing integrin, Sns, and actin. This suggests that the amount of integrin expressed is integral in both the formation of the SD and the actin cytoskeleton. Moreover, the lack of any of the components—integrin, SD, actin—can result in the cytoplasmic aggregation of all three. The process behind this remains unclear and will be a subject for future studies.

Four actin-encoding genes were expressed in nephrocytes, and each played a significant role in its cytoskeleton. While loss of any of the actin genes severely disrupted the SD structure and reduced its function, silencing of individual actin genes led to different phenotypes, suggesting that even though their actin proteins have highly similar amino acid sequences, the subtle differences among them leads to distinct roles in actin cytoskeleton formation. Loss of *Act5C* led to completely absent nephrocytes, suggesting that Act5C is a fundamental component of the actin cytoskeleton, crucial for nephrocyte development. Deficiency for any of the other three actin genes did not lead to nephrocyte loss, but did result in variations in cell size, mis-localized SD components, SD disruption, and actin stress fiber formation; this suggests that they have important, albeit different, roles in shaping the nephrocyte cytoskeleton. However, the lost nephrocytes due to *Act5C* knockdown, returned and were largely functional following expressing of *Act42A* or *Act87E*, indicating significant partial functional redundancy. The genes associated with nephrotic syndrome are highly conserved and many have been functionally validated in fly models, including the large portion that encode actin cytoskeleton-related and SD components [14,15]. Therefore, a better understanding of the nephrocyte actin cytoskeleton and its interacting partners, such as the SD and integrin, is essential to unravel the pathomechanisms that underly genetic mutations nephropathy.

## Data Availability

All source data for RNA-seq, including sequence reads and expression matrices, have been deposited in NCBI’s Gene Expression Omnibus and are accessible through GEO accession numbers: embryonic RNA-seq [35], GSE168774; and, adult low input RNA-seq, GSE266297 (see Section 2). All other relevant data can be found within the article and its supplementary information. The materials that support the findings of this study are available from the corresponding authors upon reasonable request.

## Supplementary Materials

The following supporting information can be downloaded at: https://www.mdpi.com/article/10.3390/cells13161350/s1, Figure S1: Knockdown of nephrocyte actin genes results in disrupted nephrocyte number and size; Figure S2: Act5C required for maintaining the cytoskeleton and SD structures; Figure S3: Amino acid alignment for Actin proteins with genes expressed in *Drosophila* nephrocytes or human podocytes; Figure S4: SD structure and function depend on integrin; Figure S5: Actin cytoskeleton, integrin, and SD structures are interdependent.
