# Supplementary material for: Actin Cytoskeleton and Integrin Components Are Interdependent for Slit Diaphragm Maintenance in Drosophila Nephrocytes
Source: Cells. 2024 Aug 14;13(16):1350. doi: 10.3390/cells13161350 (PMC11352372; doi:10.3390/cells13161350)

SUPPLEMENTARY FILES

Actin cytoskeleton and integrin components are interdependent for slit diaphragm maintenance in *Drosophila* nephrocytes — Delaney et al.

SUPPLEMENTARY METHODS

Drosophila husbandry

Fly lines were maintained on a standard diet (Meidi Laboratories, MD), at 25°C under a 12h:12h light:dark cycle. *Drosophila* stocks were obtained from the Bloomington Drosophila Stock Center (BDSC, IN): UAS-*Act5C*-IR (BDSC_42651), UAS-*Act42A*-IR (BDSC_50625), UAS-*Act57B*-IR (BDSC_31551), UAS-*Act87E*-IR (BDSC_42652), UAS-*Act5C-GFP* (BDSC_9258), UAS-*Act42A-GFP* (BDSC_9251), UAS-*Act57B-GFP* (BDSC_9256), UAS-*Act87E-GFP* (BDSC_9249), UAS-*GFP-Piezo* (BDSC_58773), UAS-*mew*-IR (BDSC_44553), UAS-*mys*-IR (BDSC_27735), UAS-*sns*-IR (BDSC_64872), UAS-*pyd*-IR (BDSC_28920), tub-Gal80^{ts}/TM2 (BDSC_7017), tub-Gal80^{ts}; TM2/TM6B (BDSC_7108), w^{1118} (BDSC_3605); and the Vienna Drosophila Resource Center (VDRC, Vienna, Austria): UAS-*Act5C*-IR (VDRC_101438), UAS-*Act42A*-IR (VDRC_104731), UAS-*Act57*B-IR (VDRC_102129), and UAS-*Act87E*-IR (VDRC_102480). The following are in-house fly stocks: *Hand-GFP*, *hs-Flp*^{122}; UAS-*Flp*; *Act5C*>*stop*>Gal4, UAS-*GFP* [1]; and If/CyO.

Generation of *Drosophila Klf15*-Gal4 line

To generate the *Klf15*-Gal4 transgenic line, a 2.1 kb *Klf15* promoter region was PCR amplified and cloned into the pPTGAL vector between the EcoRI and BamHI restriction sites. The plasmid was sequence verified. Microinjection was performed by Rainbow Transgenic Flies (CA).

Knock-in mRuby3 tag at the C-terminal of Sns

Transgenic *sns*-mRuby3 flies carry an mRuby3 tag at the C-terminal of endogenous Sns. The line was commercially generated by FunGene (Jiangsu, China). SnsKI-sg1 and SnsKI-sg2 gRNAs were generated using in vitro transcription (T7 RiboMAX kit; P1320; Promega, WI). The transcripts were purified by phenol-chloroform extraction and isopropanol precipitation. Cas9 mRNA in vitro transcription was carried out using plasmid MLM3613 (plasmid 42251; Addgene, MA), while the pBluescript SK vector (pBS) was used as the backbone for donor plasmid construction. Using genomic DNA of the injection stock, the 5' and 3' homology arms were amplified and linked to the pBS backbone (Gibson assembly kit; E2611L; NEB, MA). This pBS-*sns*-arm was linearized by PCR and linked to the mRuby3 cassette (Gibson assembly kit; E2611L; NEB, MA), resulting in the final donor construct: pBS-*sns*-mRuby3. The gRNA, Cas9 mRNA, and donor pBS-*sns*-mRuby3 were injected into w^{1118} flies. In house, PCR was performed to validate the mRuby3 insertion.

Immunochemistry

Female adult (1-day-old) flies were rinsed in 95% ethanol and transferred to 1xPBS. The *Drosophila* abdomen was cut open to remove intestine, Malpighian tubules, fat bodies, and ovaries. The resulting dorsal cuticles of the abdomen (with nephrocytes and heart tube attached) were fixed in 4% PFA for 1 hr at room temperature. The specimens were washed three times in 1xPBST for 15 min (0.2% Triton-x 100 in 1xPBS), then blocked in blocking buffer (1% bovine serum albumin in 1xPBST) for 1 hr. Primary antibodies were diluted in the blocking buffer and incubated overnight at 4°C, then washed in 1xPBST three times for 15 min each, followed by incubation with secondary antibodies for 2 hrs at room temperature. The samples were then incubated with phalloidin (Alexa Fluor 488 phalloidin; 1:100; A12379; Invitrogen, CA) diluted in blocking buffer for 4 days at 4°C. The following antibodies were used: Mouse monoclonal anti-Pyd (1:100; RRID:AB_2618043; Developmental Studies Hybridoma Bank, IA), anti-Mys (1:100;

CF.6G11, RRID_ AB_528310; Developmental Studies Hybridoma Bank, IA), and goat anti-mouse Alexa Fluor 488 (1:500; A11011, RRID:AB_143157; Invitrogen, CA). DAPI (0.5 mg/ml in PBST; D1306; Thermo-Fisher-Scientific, MA) was used to visualize the nuclei. Following antibody incubations, the samples were washed in 1xPBST three times for 15 min each, once with 1xPBS for 15 min, then mounted using Vectashield antifade mounting media (H-1000; Vector Laboratories, CA). Images were obtained using a ZEISS LSM900 confocal microscope with 63× Plan-Apochromat 1.4 N.A. oil objective under Airyscan mode and ZEN blue edition (version 3.0) acquisition software. Representative imaged have been shown in the figures. For quantitative comparison of fluorescence intensities, settings for the control were chosen to avoid oversaturation (using range indicator in ZEN blue), then applied across images for all samples within an assay. Image J [2] was used for image processing (version 2.9.0/1.53t; National Institutes of Health, MD).

RNA-seq embryonic and adults nephrocytes

Embryonic RNA-seq data (GSE168774; can also be accessed through the Single Cell Portal at https://singlecell.broadinstitute.org/) was carried out previously [3]. For the adult low input RNA-seq (GSE266297), the flies (*Hand*-GFP; *Klf15*-Gal4, UAS-RFP/CyO) were maintained on a standard diet (Meidi Laboratories, MD), at 25°C under a 12h:12h light:dark cycle. Nephrocytes from adult flies (4-day-old females; 3 replicates of 200 flies each) were dissected in artificial hemolymph (108 mM Na^+, 5 mM K^+, 2 mM Ca^{2+}, 8 mM $MgCl_2$, 1 mM NaH_2PO_4, 4 mM $NaHCO_3$, 10 mM sucrose, 5 mM trehalose, and 5 mM HEPES; pH 7.1) at room temperature. Large, GFP-and RFP-positive cells were sorted by flowcytometry (BD Aria II; University of Maryland Greenebaum Comprehensive Cancer Center Flow Cytometry Shared Service). following RNA was collected and cDNA libraries were prepared using the SuperScript IV Single Cell/Low-Input cDNA PreAmp Kit (Thermo-Fisher-Scientific, MA). Then, sequencing on a NovaSeq platform (Illumina, CA) was carried out by Psomagen (Rockville, MD). We mapped the short reads on to the *Drosophila*

genome using STAR aligner 2.7.5c [4],then quantified the RNA-seq reads using RSEM 1.3.3 [5]; based on *Drosophila* gene annotation 6.28 from FlyBase (Berkeley Drosophila Genome Project (BDGP) Release 6).

Nephrocyte number and size quantifications

Nephrocytes from 1-day-old adult female flies were dissect in 1xPBS, followed by fixation (1 hr) in 4%PFA, and imaged using a ZEISS LSM900 confocal microscope 20× Plan-Apochromat 0.8 N.A. air objective. The number of nephrocytes present in the image were then counted, and cell sizes were determined using the area measurement function using Fiji software [2] (version 2.9.0; National Institutes of Health, MD).

10 kD dextran or 70 kD dextran uptake

Nephrocyte functional assays were performed ex vivo at room temperature. *Drosophila* females (1-day-old adults) were dissected in Schneider's Drosophila Medium (Thermo-Fisher-Scientific, MA), then incubated for 20 min in a 10 kD Texas Red-dextran solution (0.05 mg/mL; D1828; Invitrogen, CA) in Schneider's Drosophila Medium (Thermo-Fisher-Scientific, MA), or alternatively, incubated for 1 min in a 70 kD Texas Red-dextran solution (0.25 mg/mL; D1864; Invitrogen, CA) in Schneider's Drosophila Medium (Thermo-Fisher-Scientific, MA). Following dextran uptake, the specimens were washed with Schneider's Drosophila Medium (Thermo-Fisher-Scientific, MA) twice, then fixed using 4% paraformaldehyde (PFA) for 60 min. Finally, the fixed specimens were washed three times for 5 min each with 1x phosphate buffered saline (1xPBS; pH 7.4) and mounted using Vectashield antifade mounting medium (H-1000; Vector Laboratories, CA). Images were obtained using a ZEISS LSM900 confocal microscope with 20× Plan-Apochromat 0.8 N.A. air objective and ZEN blue edition (version 3.0) acquisition software. For quantitative comparison of fluorescence intensities, settings for the control were chosen to avoid oversaturation (using range indicator in ZEN blue), then applied across images for all samples

within an assay. Image J [2] was used for image processing (version 2.9.0/1.53t; National Institutes of Health, MD).

Sns-mRuby3 and phalloidin quantifications

Since both *Klf15*-Gal4 and *sns*-mRuby3 are located on the 2nd chromosome, *Klf15*-Gal4 female virgin flies were crossed with *sns*-mRuby3 male flies, the progeny female virgins were crossed with If/CyO to create a stable stock of [*Klf15*-Gal4, *sns*-mRuby3/CyO]. These female virgins were then crossed with w^{1118}, *Piezo*-IR, *Act5C*-IR #1, *Act5C*-IR #2, *Act42A*-IR #1, *Act42A*-IR #2, *Act57B*-IR #1, *Act57B*-IR #2, *Act87E*-IR #1, *Act87E*-IR #2, *mew*-IR (encodes α-integrin subunit), or *mys*-IR (encodes β-integrin subunit). The nephrocytes from 1-day-old females were then stained according to the immunochemistry protocol detailed above and imaged using the ZEISS LSM900 confocal microscope with 63× Plan-Apochromat 1.4 N.A. oil objective under Airyscan mode and ZEN blue edition (version 3.0) acquisition software. For stress fibers and SD internalization, the number of nephrocytes with stress fibers (any cytoplasmic actin structure, based on phalloidin stain) were manually counted. Nephrocytes numbers analyzed were 13 for control, 39 for *Act42*-RNAi, 52 for *Act57B*-RNAi, and 51 for *Act87E*-RNAi flies. To determine internalized Sns (based on the presence of distinct cytosplasmic Sns-mRuby) nephrocytes were manually counted: 24 for control, 51 for *Act42A*-RNAi, 36 for *Act57B*-RNAi, and 33 for *Act87E*-RNAi flies. For quantitative comparison of fluorescence intensities, settings for the control were chosen to avoid oversaturation (using range indicator in ZEN blue), then applied across images for all samples within an assay. Image J [2] was used for image processing (version 2.9.0/1.53t; National Institutes of Health, MD). The average number of slit diaphragm lines (visualized by Sns-mRuby) were depicted via the Plot Profile software by utilizing a small section across the central area of the cortical membrane region. Nephrocytes from RNAi (-IR) flies were compared to SD patterns in control nephrocytes.

Tissue mosaic analysis

Flp-out clone [6] induction was performed as described previously [1]. In brief, *hs-Flp*^{122}; UAS-*Flp*; *Act5C*>*stop*>Gal4, UAS-*GFP* female virgins were crossed with α-*integrin*-IR and β-*integrin*-IR males. The embryos were collected within an eight-hour time window. At 24 hrs after larval hatching, a 10-minute heat shock was performed in a 37°C water bath, following which the larvae were maintained at 25°C. One-day-old female adults were subjected to 10 kD dextran functional assay (described above); GFP-positive nephrocyte clones and their neighboring nephrocytes were analyzed.

TARGET assay

Klf15-Gal4, *sns*-mRuby3 virgin female flies, were initially crossed with *tub*-Gal80^{ts}/TM2, located on chromosome 3. These virgin female flies were then crossed with *Klf15*-Gal4;*sns*-mRuby/*Act5C*-IR males, resulting in [*Klf15*-Gal4, *sns*-mRuby3/+; *tub*-Gal80^{ts}/*Act5C*-IR] flies. TARGET [7,8]: These flies were kept at 18°C until their offspring had reached 1-day adults. This allows for the Gal80 to be activated, which represses Gal4 activity. The 1-day-old adults were then transferred to 29°C for either one or two days, this temperature Gal80 gets inactivated, allowing the Gal4 to become active and knockdown the *Act5C* gene in the nephrocytes. Following this temperature switch, the flies were assayed and imaged as described above.

Actin protein sequence comparisons

Protein sequence were obtained from FlyBase [9,10] (version 2023_05: Dmel 6.54) for *Drosophila* and UniProt [11] (release 2023_04) for human, then aligned using the Multiple Sequence Alignment (MSA) package [12] in R [13] (version 4.3.1) using the Clustal Omega algorithm [14]. Phylogenetic analysis were based on protein sequences and carried out using the Neighbor-Joining Tree

Estimation function from the Analyses of Phylogenetics and Evolution (APE) package [15] in R [13] (version 4.3.1).

Statistical analysis

Fiji software [2] (version 2.9.0; National Institutes of Health, MD) was used to process the confocal images and to quantify the relative fluorescence intensity. The data sets were tested for normality using the Shapiro-Wilk test. Normally distributed data were analyzed by a two-tailed Student's t-test, a one-way ANOVA corrected with Tukey, or by a two-way ANOVA with Sidak correction. Non-normally distributed data were analyzed by a Mann-Whitney U test or a Kruskal-Wallis H test. $P < 0.05$ was considered significant. The data sets were plotted using GraphPad Prism9 software (version 9.5.1). The figures were arranged using Adobe Illustrator software (version 2022 26.2.1).

REFERENCES

1. Duan J, Zhao Y, Li H, Habernig L, Gordon MD, Miao X, et al.: Bab2 Functions as an Ecdysone-Responsive Transcriptional Repressor during Drosophila Development. *Cell Rep.* 32: 107972, 2020
2. Schneider CA, Rasband WS, Eliceiri KW: NIH Image to ImageJ: 25 years of image analysis. *Nat. Methods* 9: 671–675, 2012
3. Huang X, Fu Y, Lee H, Zhao Y, Yang W, van de Leemput J, et al.: Single-cell profiling of the developing embryonic heart in Drosophila. *Development* [Internet] 150: 2023 Available from: http://dx.doi.org/10.1242/dev.201936
4. Dobin A, Davis CA, Schlesinger F, Drenkow J, Zaleski C, Jha S, et al.: STAR: ultrafast universal RNA-seq aligner. *Bioinformatics* 29: 15–21, 2013

5. Li B, Dewey CN: RSEM: accurate transcript quantification from RNA-Seq data with or without a reference genome. *BMC Bioinformatics* 12: 323, 2011
6. Struhl G, Basler K: Organizing activity of wingless protein in Drosophila. *Cell* 72: 527–540, 1993
7. Matsumoto K, Toh-e A, Oshima Y: Genetic control of galactokinase synthesis in Saccharomyces cerevisiae: evidence for constitutive expression of the positive regulatory gene gal4. *J. Bacteriol.* 134: 446–457, 1978
8. McGuire SE, Le PT, Osborn AJ, Matsumoto K, Davis RL: Spatiotemporal rescue of memory dysfunction in Drosophila. *Science* 302: 1765–1768, 2003
9. Thurmond J, Goodman JL, Strelets VB, Attrill H, Gramates LS, Marygold SJ, et al.: FlyBase 2.0: the next generation. *Nucleic Acids Res.* 47: D759–D765, 2019
10. Larkin A, Marygold SJ, Antonazzo G, Attrill H, Dos Santos G, Garapati PV, et al.: FlyBase: updates to the Drosophila melanogaster knowledge base. *Nucleic Acids Res.* 49: D899–D907, 2021
11. UniProt Consortium: UniProt: The universal protein knowledgebase in 2023. *Nucleic Acids Res.* 51: D523–D531, 2023
12. Bodenhofer U, Bonatesta E, Horejš-Kainrath C, Hochreiter S: msa: an R package for multiple sequence alignment. *Bioinformatics* 31: 3997–3999, 2015
13. R Core Team (2021), R Foundation for Statistical Computing, Vienna, Austria: R: A language and environment for statistical computing [Internet]. Available from: https://www.R-project.org/
14. Sievers F, Wilm A, Dineen D, Gibson TJ, Karplus K, Li W, et al.: Fast, scalable generation of high-quality protein multiple sequence alignments using Clustal Omega. *Mol. Syst. Biol.* 7: 539, 2011
15. Paradis E, Schliep K: ape 5.0: an environment for modern phylogenetics and evolutionary analyses in R. *Bioinformatics* 35: 526–528, 2019

SUPPLEMENTARY FIGURES

Supplementary Figure S1. Knockdown of nephrocyte actin genes results in disrupted nephrocyte number and size

Representative images of heart tubes (dashed outline) and adjacent nephrocytes (1-day-old, female) from *Klf15*-Gal4, *Hand*-GFP flies crossed with *Act5C*-IR, *Act42A*-IR, *Act57B*-IR, and *Act87E*-IR (RNAi) lines at 25°C. Control (*Klf15*-Gal4, *Hand*-GFP>*w*^{1118}). Asterisks indicate missing nephrocytes. Scale bar: 50 μm.

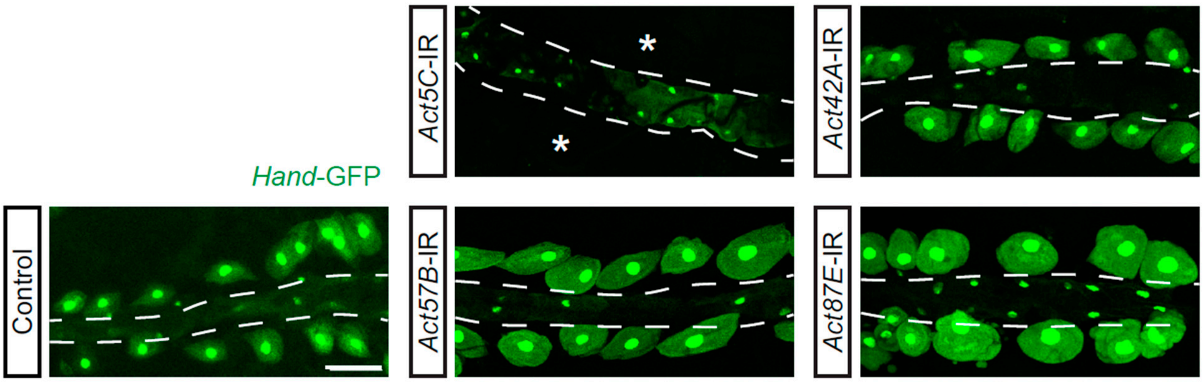

Supplementary Figure S2. Act5C required for maintaining the cytoskeleton and SD structures

(A) Diagram depicts the *Drosophila* Gal4/Gal80 system (TARGET [36,37]), which was used for temporal control of *Act5C*-IR expression, turned transcription on or off for either 1 day or 2 days. *Klf15*, Kruppel-like factor 15 nephrocyte-specific driver; *Tub*, Tubulin endogenous driver. **(B, C, D)** Representative confocal images (cortical and medial planes) of nephrocytes from control condition (1-day-old, female; B), *Act5C*-IR (1 day; C), and *Act5C*-IR (2 days; D) from the Gal4/Gal80 in (A). Phalloidin stains F-actin. Dashed line outlines the nephrocyte. Scale bars:

(cortical) 20 μm; (cortical detail) 2 μm; (medial) 20 μm; (medial detail) 2 μm.

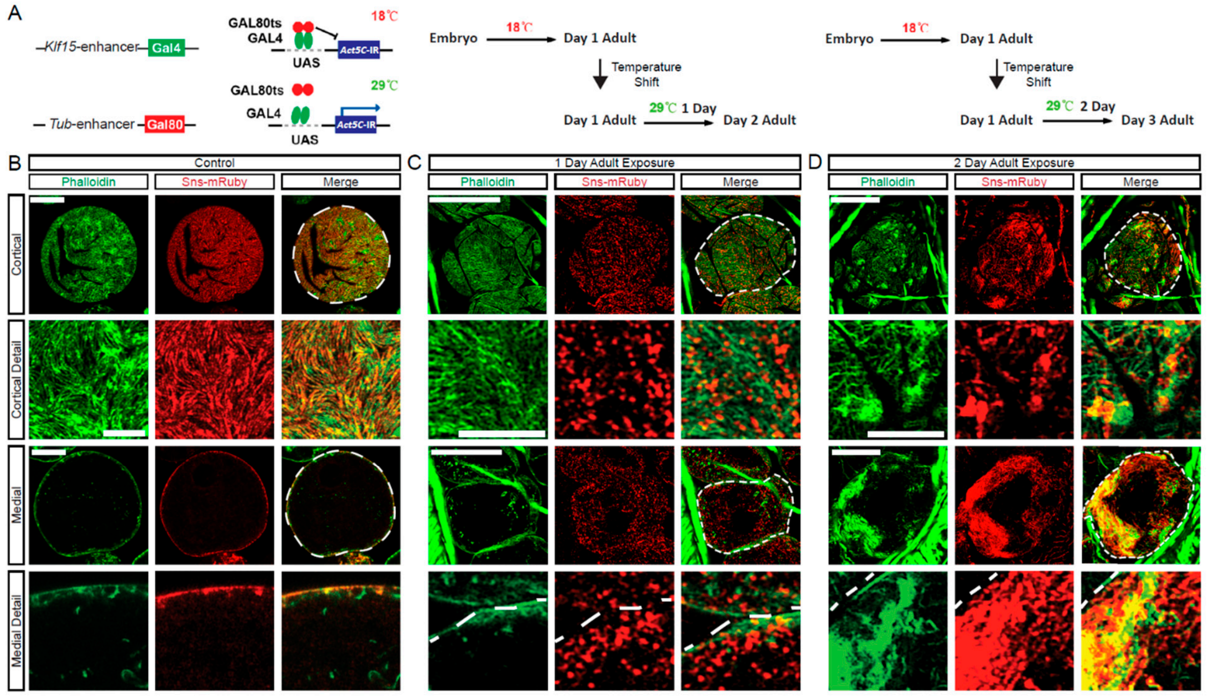

Supplementary Figure S3. Amino acid alignment for Actin proteins with genes expressed in *Drosophila* nephrocytes or human podocytes

(A) In blue, shared amino acids; in pink, different amino acids yet with similar properties; in white, amino acid differences; and, dots indicate alignment gaps. Fly protein sequences obtained from FlyBase [38,39] (version 2023_05: Dmel 6.54). Human protein sequences obtained from UniProt [40] (release 2023_04). **(B)** Diagram for the four actin proteins expressed in nephrocytes. Amino acid differences indicated in yellow are between the two groups (group 1, Act5C and Act42A; group 2, Act57B and Act87E) of more distantly related actin proteins, those in orange are between two actin proteins within a group. **(C)** Phylogenetic tree for all *Drosophila* actin proteins (based on data from FlyBase).

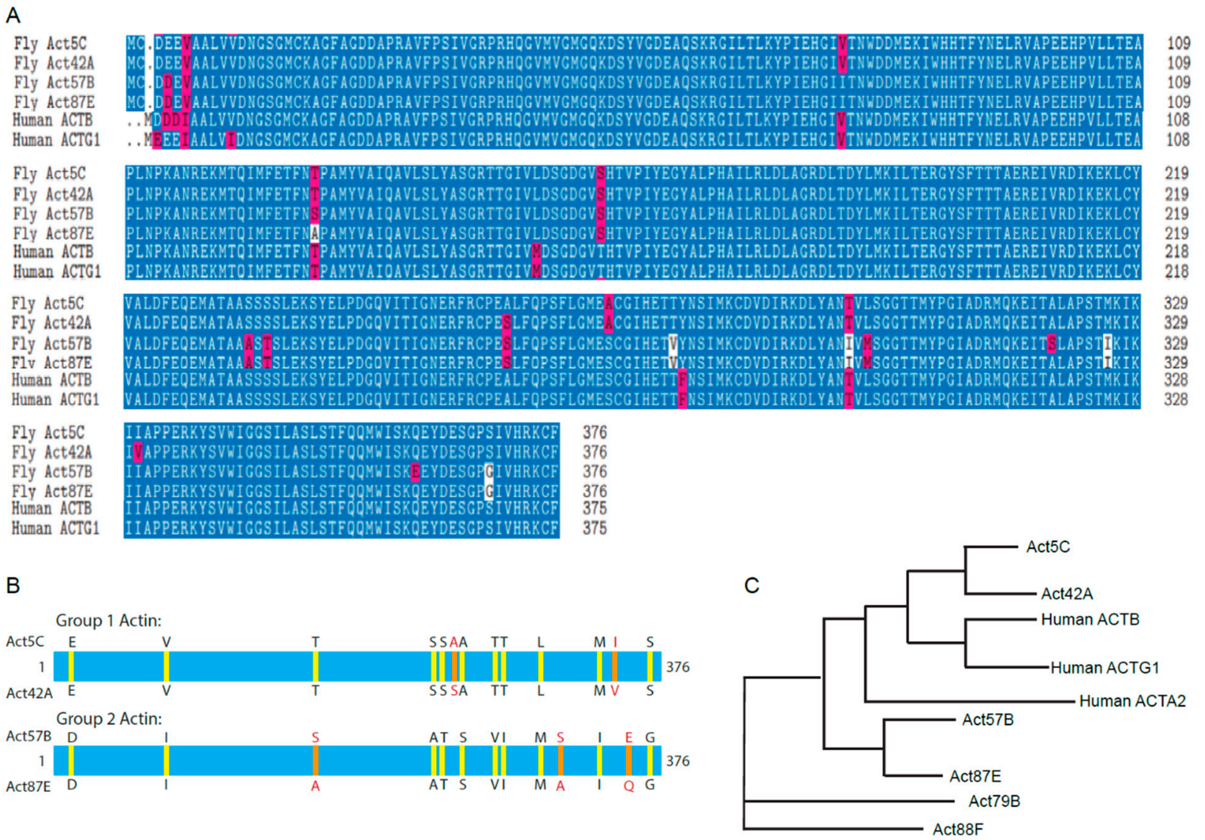

Supplementary Figure S4. SD structure and function depend on integrin

(A) Diagram depicts the flp-out system which uses heat shock to generate clones with Gal4 expression driven by the *Act5C* promoter. The Gal4 switches on both UAS-GFP and UAS-*integrin*-IR, thus green clones are deficient for integrin whereas neighboring (non-green clones) serve as controls. **(B)** Representative images of 10 kDa dextran uptake in the flp-out clone as compared to its control neighbors for nephrocytes from *alpha-integrin* (*mew*-IR) and *beta-integrin* (*mys*-IR) RNAi *Drosophila* lines (1-day-old, female). Scale bar: 50 μm. **(C)** Quantification of the relative fluorescence intensity of the 10 kDa dextran uptake by nephrocytes of the flp-out in (D). Statistical analysis: two-tailed Student's t-test corrected with Welch; *, P<0.05; **, P<0.01. (D**)** Representative confocal images of the cortical surface of nephrocytes from control (*Klf15*-Gal4, *sns*-mRuby3/+), *alpha-integrin*-IR (*mew* RNAi), and *beta-integrin*-IR (*mys* RNAi) flies (1-day-old, females). Dashed line outlines the nephrocyte. Boxed areas in (D) shown magnified in (Figure

5C). Scale bar: 10 μm. **(E)** Representative confocal images of the medial plane of nephrocytes from control (*Klf15*-Gal4, *sns*-mRuby3/+), *alpha-integrin*-IR (*mew* RNAi), and *beta-integrin*-IR (*mys* RNAi) flies (1-day-old, females). Dashed line outlines the nephrocyte. DAPI stain used to visualize the nucleus. Boxed areas in (E) shown magnified in (Figure 5D). Scale bar: 10 μm.

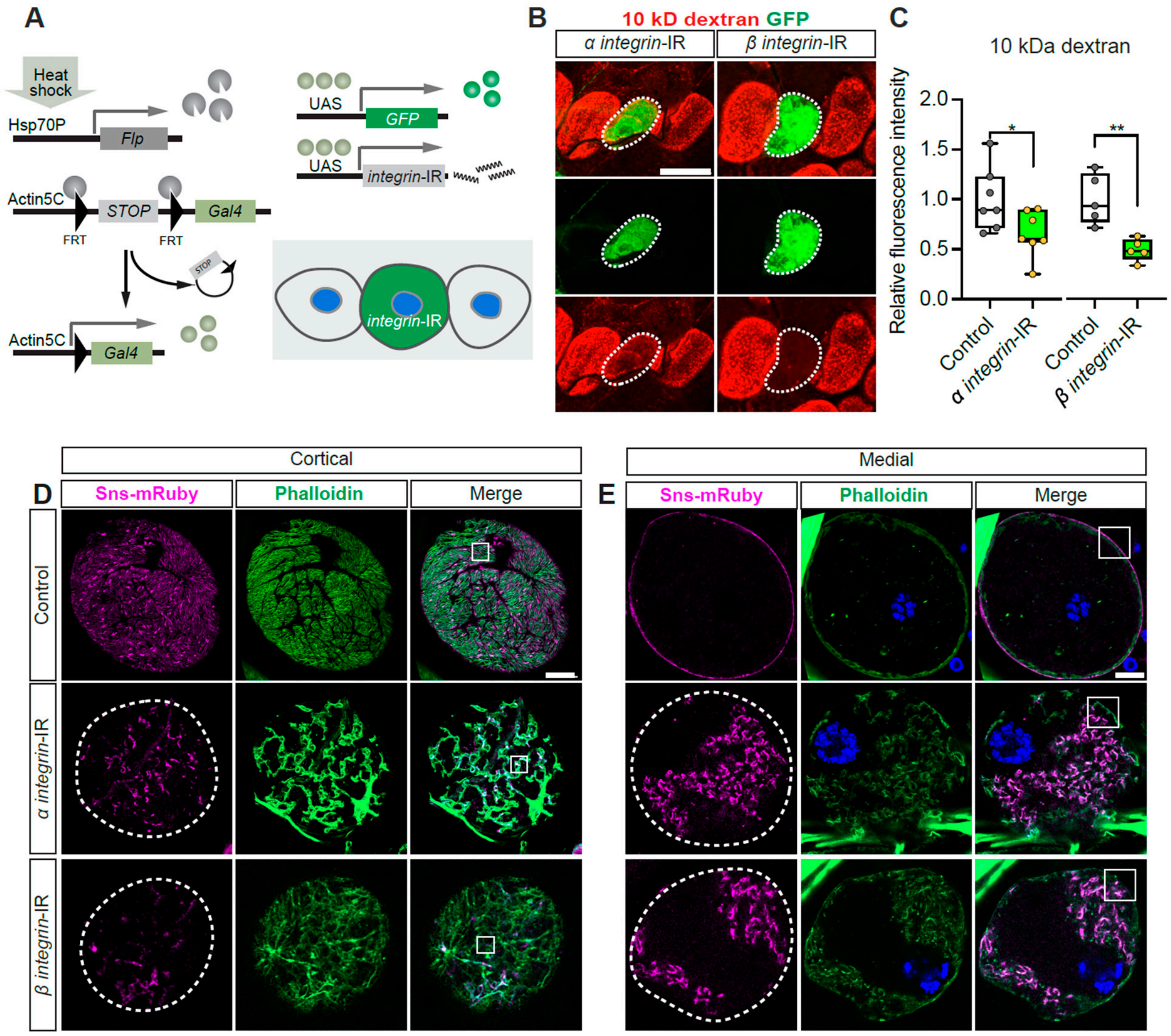

Supplementary Figure S5. Actin cytoskeleton, integrin, and SD structures are interdependent

(A) Representative confocal images of cortical surface of nephrocytes from control (*Klf15*-Gal4, *sns*-mRuby3/+) and *Act87E*-IR (RNAi) flies (1-day-old, female). Boxed area in (A) shown

magnified in (Figure 6A). Scale bars: 10 μm. **(B)** Representative confocal images of medial view of nephrocytes from control (*Klf15*-Gal4, *sns*-mRuby3/+) and *Act87E*-IR (RNAi) flies (1-day-old, female). Boxed area in (B) shown magnified in (Figure 6B). Scale bars: 10 μm. **(C)** Representative confocal images of cortical surface of nephrocytes from control *(Klf15*-Gal4, *sns*-nRuby3/+), *pyd*-IR (RNAi), and *sns*-IR (RNAi) flies (1-day-old, female). Boxed area in (C) shown magnified in (Figure 6C). Scale bars: 10 μm. **(D)** Representative confocal images of medial views of nephrocytes from control *(Klf15*-Gal4, *sns*-mRuby3/+), *pyd*-IR (RNAi), and *sns*-IR (RNAi) flies (1-day-old, female). Boxed area in (D) shown magnified in (Figure 6D). Scale bars: 10 μm.

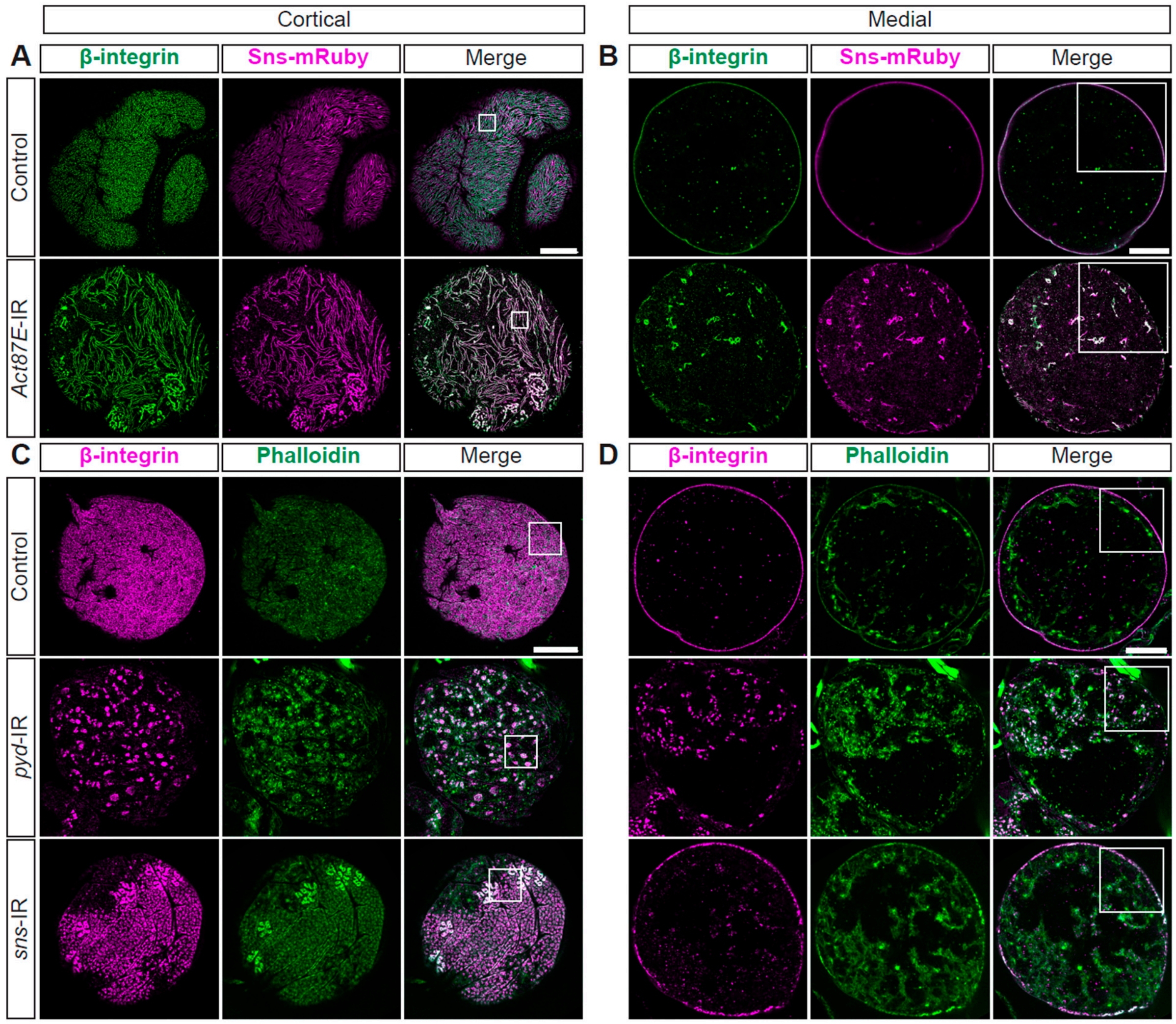

Supplement: Supplementary file 1 [file cells-13-01350-s001.zip › cells-3138758-supplementary.pdf]
